# Erector spinae plane block versus thoracic paravertebral block for the prevention of acute postsurgical pain in breast cancer surgery: A prospective observational study compared with a propensity score-matched historical cohort

**DOI:** 10.1371/journal.pone.0279648

**Published:** 2022-12-30

**Authors:** Antoine Premachandra, Xiaomeng Wang, Mary Saad, Sahar Moussawy, Roman Rouzier, Aurélien Latouche, Aline Albi-Feldzer

**Affiliations:** 1 Department of Anaesthesiology, Institut Curie, PSL Research University, Saint-Cloud, France; 2 INSERM, U900, Institut Curie, PSL Research University, Saint-Cloud, France; 3 Department of Research and Development, Sanofi, Chilly Mazarin, France; 4 Conservatoire National des Arts et Métiers, Paris, France; 5 Department of Surgical Oncology, Centre François Baclesse, Caen, France; Sapienza University of Rome: Universita degli Studi di Roma La Sapienza, ITALY

## Abstract

**Background:**

Preventing acute postsurgical pain (PSP) following breast cancer surgery is a major issue. Thoracic paravertebral block (TPVB) has been widely studied for this indication. Erector spinae plane block (ESPB) has been assumed to be effective. We aimed to compare the efficacy and safety of ESPB over TPVB in preventing acute PSP.

**Methods:**

In this prospective observational study, 120 patients admitted for unilateral major oncologic breast surgery received T2/T3 ESPB (ropivacaine 0.75%, 0.35 ml.kg^-1^), and 102 were analysed. Then, the ESPB cohort was compared to a TPVB cohort from the experimental arm of a randomized controlled study with the same protocol (NCT02408393) using propensity score matching analysis. The primary outcome was the need for morphine consumption in the PACU. Secondary outcomes were the morphine total dose, the incidence of ESPB and TPVB complications, and discontinuous visual analogue scale measurement trends at rest and at mobilization in the 24 hours after surgery.

**Results:**

A total of 102 patients completed the study between December 2018 and August 2019. Propensity score matching formed 94 matched pairs. The proportion of morphine titration in the PACU was higher in the ESPB group than in the TPVB group (74.5% vs. 41.5%, p<0.001), with a between-group difference of 33.0% (95% CI [19.3%, 46.7%]). No ESPB-related complications were observed.

**Conclusion:**

ESPB is less effective in preventing morphine consumption in the PACU than TPVB. Our findings do not support the use of ESPB as the first-line regional anaesthesia for major breast cancer surgery. Randomized trials comparing ESPB and TPVB are needed.

## Introduction

The incidence of acute postsurgical pain (PSP) following breast cancer surgery is as high as 70% [1]. Acute PSP impacts quality of life and may increase the risk of chronic PSP [2,3]. Thus, any PSP reducing strategy is highly beneficial for patients.

Recent evidence suggests that regional anaesthesia (RA) in a multimodal analgesia program can efficiently minimize acute PSP and opioid consumption after breast cancer surgery [4–6]. Thoracic paravertebral block (TPVB) has been widely studied and considered the “gold standard” for major breast cancer surgery. In addition, this technique has recently been proven to be safe when performed under ultrasound guidance [1,7,8]. Erector spinae plane block (ESPB), described in 2016 as an alternative to TPVB, is an interfacial block performed in the plane of the spine erector muscles, which is more superficial than the paravertebral space [9]. Clinical and cadaveric studies suggest that ESPB may act on the ventral rami of the spinal nerves in the paravertebral space via a diffusion process through the costotransverse foramen and the costotransverse ligament [10,11]. ESPB is more advantageous than TPVB because it is a simpler, faster procedure [12], and because of easily identifiable landmarks, it may be safer because of more distant injection sites from the pleura and perimedullary space [13] (Fig 2).

Although ESPB has been shown to be efficient in preventing acute PSP after spine and thoracic surgery [14,15], the reliability of this diffusion process remains controversial, and ESPB has been proposed for breast surgery without evidence supporting its efficacy [16–18]. Moreover, the clinical relevance of its benefits has been questioned [19].

In breast surgery, ESPB has been shown to be superior to general anaesthesia alone and to placebo [16,20–23].

However, there is no large study comparing ESPB to TPVB for major breast surgery. The available studies comparing ESPB to TPVB have shown analyses of a relatively low number of patients, and their results are pooled in several meta-analyses that have shown conflicting results [24–27]. Thus, the role of ESPB in the strategy of analgesia for breast surgery is not clearly defined, and the comparison of ESPB performance with that of TPVB will provide some answers.

Hence, we conducted a prospective observational study to evaluate the safety and efficacy of ESPB with ropivacaine for minimizing morphine consumption in the postanesthesia care unit (PACU) after major breast cancer surgery. Then, we aimed to compare these results to an external control arm of TPVB by leveraging a historical cohort from a multicentric randomized trial, the MIRs03 study (NCT02408393) [1], which compared the efficacy of TPVB with ropivacaine to a thoracic paravertebral injection of saline in preventing acute and chronic PSP (Institut Curie, Saint-Cloud, France, Numéro EudraCT: 2014-002436-13). For our primary aim, we tested the hypothesis that ESPB increases the incidence of acute PSP when compared to TVPB. We performed a propensity score matching analysis to compare the endpoints of interest while accounting for between-study heterogeneity.

## Methods

### Study design

This prospective observational study was approved and registered by the Institutional Review Board (IRB) of the Curie Institute of Paris-Saint Cloud in December 2018. Information was provided orally, and oral consent was obtained. The IRB waived the need for written consent.

To allow the comparison to TPVB, we compared the outcomes of the ESPB cohort to those of patients in the experimental arm of the MIRs03 study [1].

### Patient management

We used the same inclusion criteria as MIRs03: female patients aged 18–85 years with an American Society of Anaesthesiologists status of I to III who were admitted for mastectomy with or without axillary lymph node or sentinel lymph node dissection or partial mastectomy with axillary lymph node dissection.

The exclusion criteria were as follows: male sex; life expectancy less than 2 years; active malignant disease; pregnancy; breastfeeding; bilateral surgery; ipsilateral breast surgery in the past 3 years; chronic pain; allergy to local anaesthetics (LA), steroids and morphine; reported history of substance abuse; local skin inflammation at the puncture area; and inability to comply with the protocol for any reason.

### Procedure

**Fig 1:**
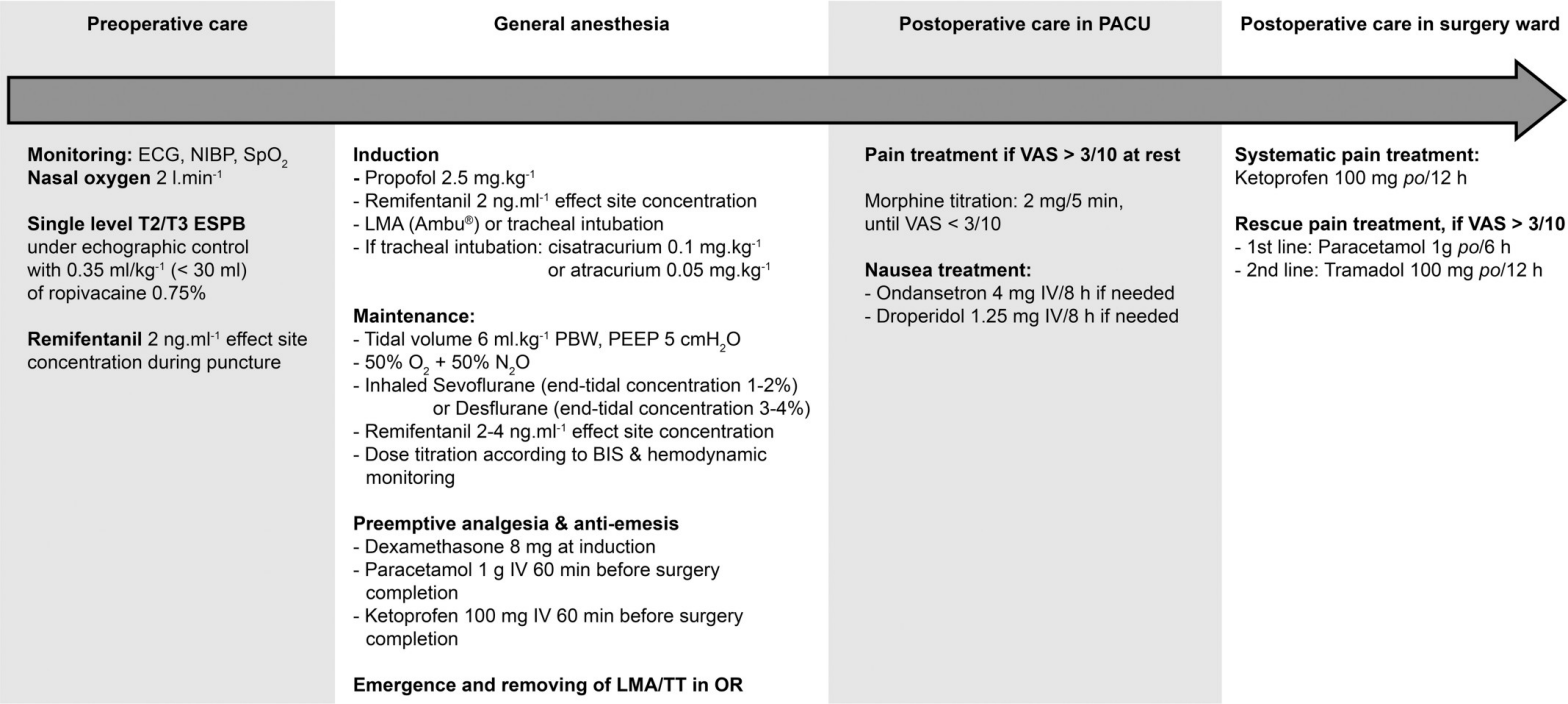
Study protocol.

The medical protocol of the ESPB study was the same as that of MIRs03 and is summarized in Fig 1.

No premedication was given.

Upon arrival at the PACU, ECG, NIBP and SpO_2_ were installed, and oxygen (2 l.min^-1^) was delivered. Patients were placed in the lateral position and received target-controlled infusion of remifentanil with a targeted 2 ng.ml^-1^ effect-site concentration.

**Fig 2:**
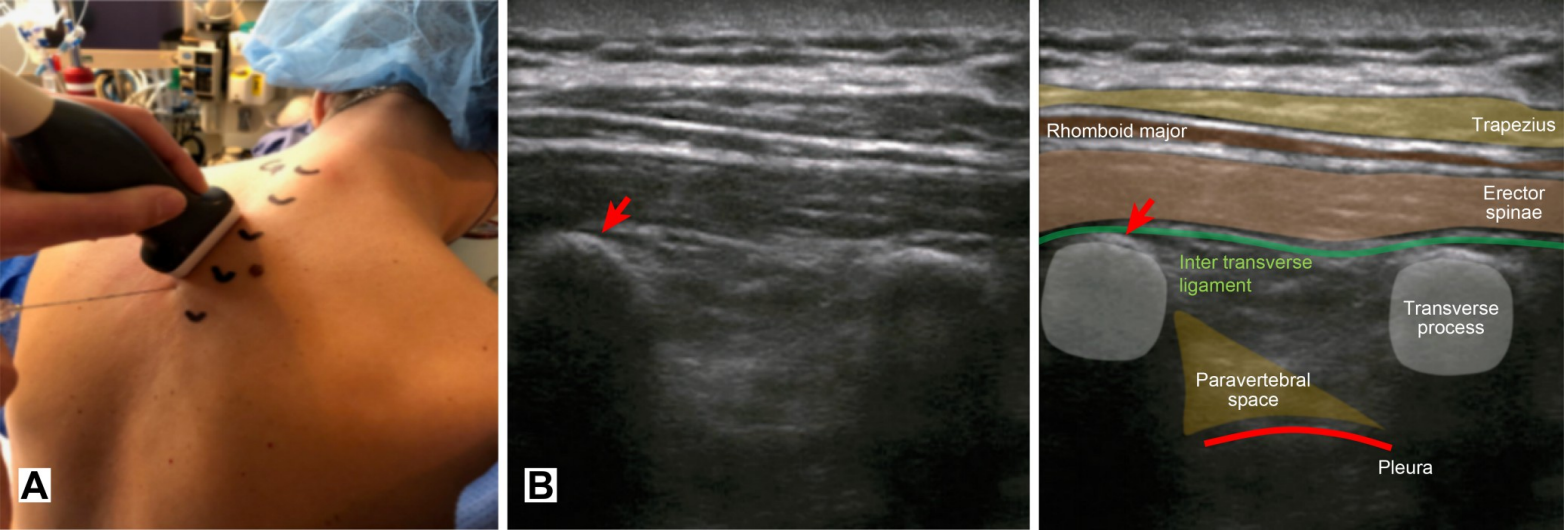
Technique description. *A*. Probe and needle placement. *B*. Echoanatomy and injection site (red arrow).

Under aseptic conditions, single-level T2 or T3 (T2/T3) ESPB was placed by senior clinicians with significant experience in thoracic wall blocks. The probe (Model Alpinion E-cube i7 with a 2–5 MHz ultrasound probe linear array L3-8H) was placed in the parasagittal plane at a 90-degree angle to the transverse process after determining the T2 and T3 transverse processes by ultrasound. The needle (22-gauge 80-mm Pajunk, SonoTAP) was advanced in the plane of the ultrasound (US) beam with the bevel oriented in a cranial direction (Fig 2). When the needle tip was positioned between the erector spinae muscle and transverse process, a hydrodissection was carried out with 1–3 ml of saline solution to confirm erector spinae muscle fascia plane dissection. Then, 0.35 ml.kg^-1^ ropivacaine 0.75% without exceeding 30 ml was injected (Fig 2).

During the MIRs03 trial, the TPVB was performed as follows: the patients were placed in the lateral position on the opposite side from surgery, and remifentanil administration was started with an IV targeted effect-site concentration objective to reach a concentration of 2 ng.ml^-1^. The second thoracic paravertebral space (T2) was scanned by ultrasonography (Model Alpinion E-cube i7 [Alpinion Medical Systems, Korea]) with a 2- to 5-MHz ultrasound probe (linear array L3-8H). The probe was positioned on the transverse plane against the spinal process. Under aseptic conditions, a 22-gauge 80-mm needle (SonoTAP [Pajunk, Germany]) was advanced in an “in-plane” direction towards the paravertebral space, immediately above the pleura and below the costotransverse ligament. The position of the needle was confirmed by the descent of the pleura when injecting 2 to 3 ml of saline solution for hydrolocalization. Then, 0.35 ml.kg^-1^ ropivacaine 0.75% was injected with intermittent negative aspiration tests every 5 ml, without exceeding a total of 30 ml or an equivalent volume of saline. Immediately after the paravertebral block injection procedure was completed in the preoperative holding area, remifentanil injection was discontinued, and the patients were transferred to the operating room 30 min later [1].

The anaesthesia management is detailed in Fig 1. After completion of the surgery, all patients were awake, breathed spontaneously and transferred to the PACU.

PSP intensity at rest and upon elevation of the arm ipsilateral to the surgery was measured upon arrival in the PACU and then every 30 minutes during the first 2 hours and every 6 hours for the first 24 hours using a VAS ranging from 0 (no pain at all) to 10 (worst imaginable pain). In the case of a resting VAS > 3/10 in the PACU, intravenous morphine titration was administered using boluses of 2 mg every 5 minutes (no upper limit of dose) until the VAS dropped ≤ 3/10. All patients stayed at least 2 h, and then they were allowed to leave the PACU if VAS was ≤ 3/10 for 30 min and the modified Aldrete Score reached at least 9. The PSP management is detailed in Fig 1.

The concentration of ropivacaine, the injected volume, the sedation and general anaesthesia protocol, the acute PSP management protocol and the postoperative nausea and vomiting (PONV) management protocol were the same as those performed with TPVB in the MIRs03 study.

### Data collection

The recorded data included age, weight and height, the type of surgery, the injected volume and dose of ropivacaine, the occurrence of ESPB-related complications, the rest and mobilization VAS measured during the hospital stay and 24 hours after surgery, the need for rescue morphine, and the overall morphine dose (mg) administered in the PACU.

The same data on TPVB were collected during the MIRs03 study. The consent given for the MIRs03 study included the reuse of data.

### Outcomes

The primary outcome was the percentage of patients who needed morphine titration in the PACU. The secondary outcomes were the total dose of morphine in the PACU, the incidence of RA complications, and discontinuous VAS measurement trends at rest and at mobilization during the first 24 hours after surgery.

### Statistical analysis

Evidence shows that approximately 25% of patients required morphine titration after TPVB [7]. The sample size was calculated based on the accuracy of the estimate of the efficacy. For an expected rate of patients requiring morphine titration of 50%, the inclusion of 106 patients produces a two-sided 95% confidence interval with a width equal to 20%.

Baseline and outcome comparisons were performed by chi-square or Fisher’s exact test for categorical variables and Student’s t test for continuous variables. The ESPB and TPVB cohorts were compared for age, body mass index (BMI), and surgery type, which are potential prognostic factors of acute PSP [28].

To balance the patient characteristics between the ESPB and TPVB cohorts, we conducted a propensity score matching analysis. The propensity scores were estimated by a multivariable logistic regression model in which the probability of receiving the intervention (ESPB vs. TPVB) was regressed conditional on age, BMI, and surgery type.

Patients were matched on the logit of the propensity score using a calliper of width equal to 0.2 of the standard deviation of the logit of the estimated propensity scores [29]. Matching was performed without replacement (i.e., each subject was available for matching only once) in a greedy manner (i.e., at each step in the matching process, the nearest TPVB subject was selected for matching to the given ESPB subject). The balance of covariates between the two arms was checked using standardized mean differences (SMDs) before and after matching. A standardized mean difference of less than 0.1 is considered to indicate a negligible difference in the mean or prevalence of a covariate between groups [29].

The risk difference in acute PSP with a 95% confidence interval was estimated as the difference between the probability of receiving morphine titration of TPVB patients and that of ESPB patients in the matched sample. The standard errors were estimated using cluster-robust standard errors to account for pair membership [30].

In addition, we built a random forest model in addition to the logistic regression model to assess the sensitivity of the matching result to the propensity score estimation [31]. The random forest model was constructed using the intervention (ESPB vs. TPVB) as the output and the baseline characteristics as inputs. Matching was performed with the same parameters as described above.

Comparisons of patient characteristics of ESPB placement details according to the need for morphine titration were performed with the Mann–Whitney or Student’s t test after testing for normality with the Shapiro–Wilk test.

The outcomes and baseline characteristics of patients were compared according to the injected volume (<25 ml vs. ≥25 ml).

All tests were two-sided. A P value of less than 0.05 was considered to indicate statistical significance. All analyses were conducted using R statistical software, version 4.0.2 (R Foundation for Statistical Computing, Vienna, Austria).

## Results

**Fig 3:**
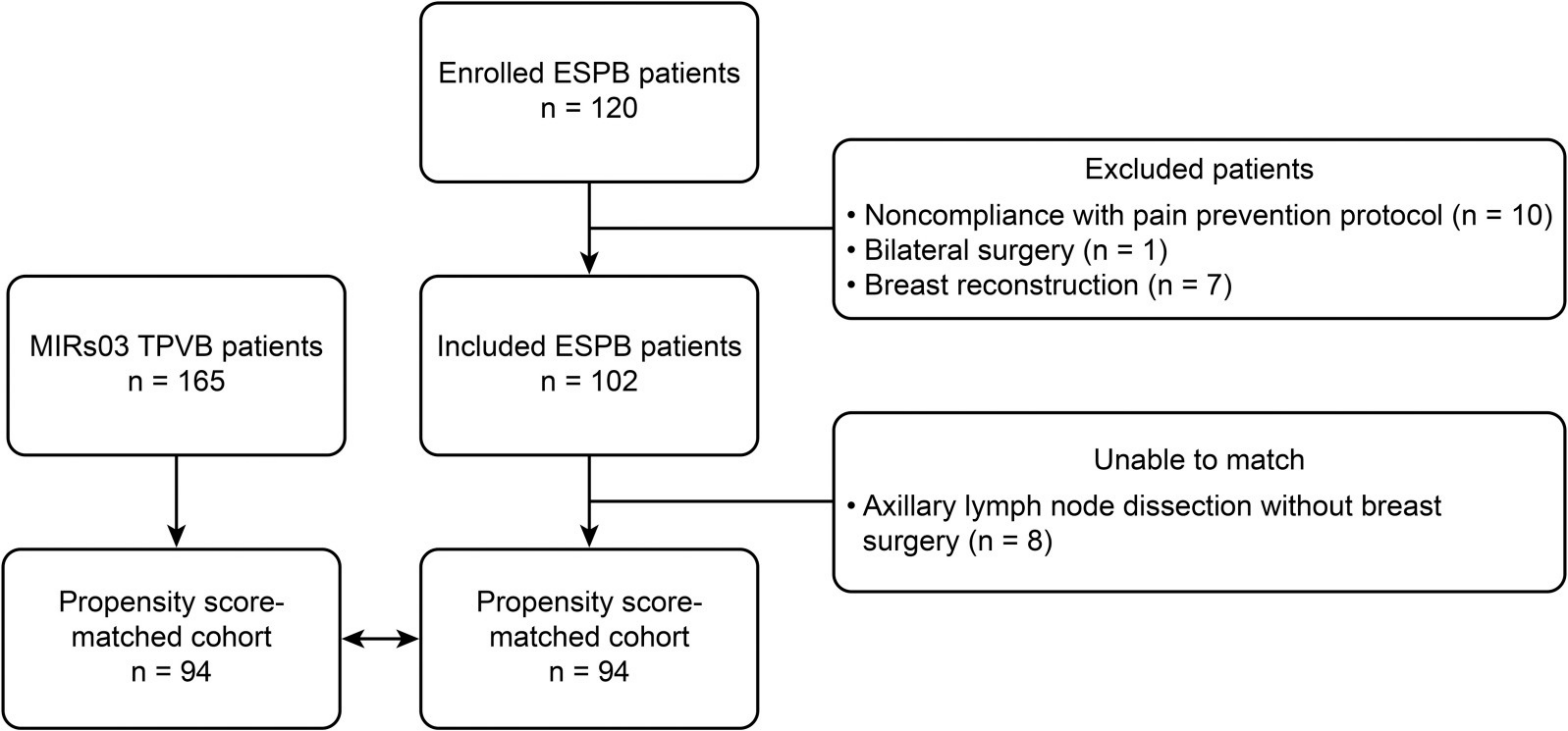
Flow chart.

In the ESPB cohort, 120 patients were enrolled between December 2018 and August 2019, and 102 patients were included in the analysis. The reasons for secondary exclusion of the 18 patients are detailed in Fig 3.

As there was no significant between-centre difference in the incidence of morphine consumption in the MIRs03 study (S4 Table), data from all 178 patients in the experimental arm were employed, of which 13 patients were excluded because of missing data on morphine consumption.

The study sample consisted of 102 ESPB patients and 165 TPVB patients. There were statistically significant differences in baseline characteristics regarding breast surgery type (ESPB patients underwent more total mastectomies, p<0.001 and SMD > 0.1) and BMI (ESPB patients had lower BMI, SMD > 0.1).

Propensity score matching formed 94 matched pairs, which means that 94 of 102 ESPB patients were matched with a TPVB patient. Eight ESPB patients who received axillary lymph node dissection were thus excluded from further analysis. The distributions of the estimated propensity scores by logistic regression are presented in the Supplementary Materials (S2 Fig).

The baseline characteristics of ESPB and TPVB patients in the propensity score-matched sample are described in Table 1. The mean and prevalence of continuous and categorical variables were very similar between the two groups (all SMDs < 0.1).

**Table 1:** Baseline characteristics of patients before and after propensity score matching.

	All patients				Propensity score-matched patients			
	ESPB (n = 102)	TPVB (n = 165)	*P*	SMD	ESPB (n = 94)	TPVB (n = 94)	*P*	SMD
Age (years), mean (SD)	56.5 (12.9)	57.3 (14.1)	0.65	0.057	56.0 (12.7)	55.2 (14.3)	0.72	0.053
Weight (kg), mean (SD)	67.20 (13.83)	68.85 (13.87)	0.35	0.119	67.05 (14.08)	67.65 (13.59)	0.77	0.043
BMI (kg/m^2^), mean (SD)	25.2 (5.0)	25.8 (5.0)	0.34	0.122	25.1 (5.1)	24.9 (4.5)	0.78	0.041
Surgery, n (%)			< 0.001	0.466			0.71	0.080
Mastectomy	74 (72.5)	143 (86.7)			74 (78.7)	77 (81.9)		
Tumorectomy	20 (19.6)	22 (13.3)			20 (21.3)	17 (18.1)		
Axillary lymph node dissection	8 (7.8)	0 (0.0)			0 (0.0)	0 (0.0)		

The primary endpoint of this study was the effect of ESPB on the need for morphine titration after breast surgery in the PACU (indicated in both cohorts when VAS > 3). In the propensity score-matched sample, the percentage of patients who required morphine titration was significantly higher in the ESPB group than in the TPVB group (74.5% vs. 41.5%, p<0.001). The observed difference between the two groups was 33.0% (95% confidence interval [CI] 19.3%, 46.7%).

Regarding secondary outcomes, as shown in Table 2, among all the propensity score-matched patients, the overall morphine dose was significantly higher in the ESPB group than in the TPVB group (3.7 mg vs. 2.2 mg, p = 0.02). Among those who received morphine titration, there was no difference in the morphine dose (5.1 mg vs. 6.1 mg, p = 0.07).

**Table 2:** Primary and secondary outcomes in propensity score-matched patients.

	ESPB (n = 94)	TPVB (n = 94)	*P*
Need for morphine titration, n (%)	70 (74.5)	39 (41.5)	< 0.001
Morphine dose (mg), mean (SD)	3.7 (3.3)	2.2 (3.2)	0.02
RA placement complication incidence, n (%)	0 (0)	7 (7.4)	0.001

The mean injected volume and dose of ropivacaine in the ESPB and TVPB groups were 22.4 ml ± 4.2 vs. 23.3 ml ± 3.6 (p = 0.03) and 166 mg ± 35 vs. 174 mg ± (p = 0.07), respectively.

In the ESPB cohort, the VAS score was reported every 30 minutes in the PACU during the first 2 hours and then every 6 hours during 48 hours; the highest score was reported 30 minutes after surgery (mean VAS 3.9 ± 2.2) (S1 Fig).

No ESPB-related complications were observed. In the TPVB cohort, 5 cases of Claude Bernard Horner syndrome, 1 case of nausea and 1 case of refractory hypotension during surgery were observed (p = 0.001).

There was no significant difference in the incidence of the need for morphine between the LA volume <25 ml group and the LA volume ≥25 ml group (70.0% vs. 81.0%, p = 0.32; S2 Table).

In the sensitivity analysis, matching of propensity scores estimated by the random forest model obtained consistent results with those of the logistic regression model (S3 Fig).

## Discussion

Our study is one of the largest clinical studies in which the efficacy and safety of ESPB in preventing acute PSP after major breast cancer surgery are evaluated [25,32].

Regarding efficacy, ESPB was less likely to prevent morphine consumption in the PACU than TPVB, with an observed difference of 33%. The incidence of morphine titration was as high as 74.5% after ESPB, and the overall morphine dose was significantly higher in the ESPB group than in the TPVB group (3.7 mg vs. 2.2 mg, p = 0.02).

Although there are no placebo-controlled studies evaluating the effect of ESPB on preventing acute PSP after breast surgery, many controlled studies comparing ESPB to standard care were conducted and showed that both TPVB and ESPB were superior to their control groups. Because ESPB seems to be safer than TPVB and takes less time for novice practitioners to learn [12], many authors have proposed ESPB as a standard-of-care RA for breast surgery.

Of interest, there are some reports suggesting the efficacy of ESPB in preventing acute PSP in this indication; some of them are randomized. In three meta-analyses, researchers compared the effect of ESPB to that of TPVB, but 2 also included thoracic surgery patients [25,27]. One meta-analysis included patients undergoing total mastectomies and found no statistically significant difference in morphine consumption at 24 hours [24]. In a more recently published randomized study, researchers were unable to demonstrate the noninferiority of ESPB to TPVB in minor breast surgery [33]. All these studies were conducted on small groups of patients.

The diffusion process of LAs to the paravertebral space has been shown to be impacted by the injected volume [10,11], but it seems to be inconsistent [34].

In the present T2/T3 ESPB evaluation, the median LA-injected volume was > 20 ml, which is quite a large volume when compared to other studies. Regarding the impact of the injected volume, receiving more or less than 25 ml of LAs did not influence the morphine titration incidence (70% vs. 81%, p = 0.32). Additionally, the injected volume was similar between ESPB patients who received and those who did not receive morphine titration. Rather than testing fractionated volumes of LAs at multiple levels, we decided to use a single-site large LA volume injected at T2/T3 to reinforce a possible volume effect allowing sensory nerve roots arising from T2 to be blocked.

Moreover, the LA concentration seems to matter [17], suggesting that a large volume and high concentration should be used to increase the probability of efficacy. In this setting and considering its rapid and extensive rate of absorption [35], safety remains to be demonstrated.

With no complications reported, US-guided ESPB placement seems to be a safe technique. However, regarding the low rate of RA technique complications, including TPVB, our sample size may be insufficient to exclude the possibility of rare complications.

Overall, there are few studies comparing ESPB to TPVB, and the results are conflicting. These differences may be attributed to several factors. First, regarding the type of surgery, we only studied the analgesic effect following major breast surgery (e.g., mastectomy with or without axillary node dissection), rather than that following minor breast surgery (e.g., lumpectomy and partial mastectomy). Second, the pain treatment strategy in the PACU is as follows: the threshold to trigger morphine titration in our centre is a VAS score ≥ 3, while some authors used a VAS score ≥4 and others used patient-controlled analgesia with or without continuous infusion of opioids. Third, regarding the concentration and volume of the LAs used, we used ropivacaine 0.75%, and one may hypothesize that using a higher volume of a solution with a lower concentration may change the results.

Different limitations in this work should be noted. First, this is an observational cohort study compared with a historical group. When involving historical data, between-study differences can be a major concern [36]. In our study, the identical design (same eligibility criteria and protocol for perioperative management) of the ESPB and TPVB trials is the main advantage supporting comparable patient characteristics, intervention effects, and outcome measurements. In addition, we balanced three important prognostic factors of acute PSP (age, BMI and breast surgery type) between the groups by conducting a propensity score matching analysis. To the best of our knowledge, there were no remaining systematic differences in the baseline covariates that could be prognostic of acute PSP in propensity score-matched subjects. The completion dates of the two studies were 2018 and 2019, so we assumed that there was no substantial evolution of clinical practice. In addition, we obtained consistent results when matching propensity scores estimated by the logistic regression model and random forest model, which showed the robustness of our conclusion. On the other hand, unmeasured confounders in observational studies may cause bias. For instance, although the care protocols used in the ESPB cohort were identical to those used in the MIRs03 study, the multicentre nature of the latter could theoretically generate heterogeneity of practice. However, in the MIRs03 study, the centre had no impact on morphine consumption (S4 Table). Of note, in the MIRs03 study, patients were recruited from March 27, 2015, to June 3, 2018, and recruitment for the ESPB cohort began in December 2018. Thus, from December 2018 to August 2019, all patients admitted to our centre for major breast surgery were treated with ESPB.

Second, our primary endpoint, the incidence of the need for morphine consumption in the PACU, can be discussed. Such a criterion allows us to evaluate the effectiveness of the block in preventing low early postoperative pain peaks. To completely understand the effects of this block, other parameters could be evaluated, such as the late consumption of analgesics or late mobilization. Indeed, some authors have hypothesized a delayed diffusion of the local anaesthetic from the injection zone to the paravertebral space with spontaneous respiratory movements, which could be responsible for delayed efficiency [37]. Next, in this study, we did not evaluate functional criteria or patient satisfaction. Finally, the other RA techniques used in breast surgery, such as PECs or serratus blocks, were not evaluated in this study.

These data suggest that ESPB should not be proposed as the first-line treatment over TPVB for the prevention of acute low-peak PSP after major breast cancer surgery. However, because of these limitations, a randomized trial is necessary to confirm these results. Hence, we are conducting a multicentric double-blind randomized trial to test the non-inferiority of ESPB compared to TPBV (ER-One, NCT04827030). The process of patient inclusion has already started, and the estimated completion date is August 2023.

## Conclusions

In this comparative study using a propensity score matching analysis with a historical arm, US-guided ESPB at the T2/T3 level was not effective in preventing morphine consumption in the PACU after major breast surgery compared with TPVB.

Despite its easy implementation, the use of ESPB as the standard of care for radical breast cancer surgery is not justified over TPVB.

## Supporting information

S1 Fig VAS boxplot. Both rest and mobilization VAS peaks were encountered at 30 min of PACU stay.
(DOCX)

S2 Fig Distribution of the estimated propensity scores using a logistic regression model. The overlapping area of propensity scores of the two groups implies that there are patients who share similar propensity scores and can thus be considered matched pairs.
(DOCX)

S3 Fig Distribution of estimated propensity scores using the random forest model. S3 Fig presents the distributions of the estimated propensity scores using the random forest model, which are similar to those estimated by the logistic regression model in S2 Fig.
(DOCX)

S1 Table Baseline characteristics and injected volume according to morphine titration need status in the ESPB cohort. There was no statistically significant difference in patient characteristics or ESPB injected volume between patients who required morphine titration and those who did not. ^a^t test when the Shapiro–Wilk test and q-q plots do not reject normality. ^b^Mann–Whitney test when the Shapiro–Wilk test or q-q plots reject normality.
(DOCX)

S2 Table Baseline characteristics and outcomes according to the injected volume in the ESPB cohort. There was no significant difference in the need for morphine titration, overall morphine dosage or VAS at rest or mobilization according to BMI. VAS, visual analog scale. ^a^t test when the Shapiro–Wilk test and q-q plots do not reject normality. ^b^Mann–Whitney test when the Shapiro–Wilk test or q-q plots reject normality.
(DOCX)

S3 Table Baseline characteristics and outcomes of patients matched on propensity scores estimated by the random forest model. This table shows the comparisons of the baseline characteristics and outcomes of patients matched on propensity scores estimated by the random forest model. Ninety-five out of 102 ESPB patients were matched with a TPVB patient. Seven ESPB patients who received axillary lymph node dissection but no breast surgery were excluded from matching. Across the baseline covariates, the absolute SMDs of age and BMI were below 0.1, indicating a negligible difference. The SMD of the performed surgery type was 0.169, which slightly exceeded the preset threshold of 0.1 but was lower than the value of 0.466 before matching. The matching process created two groups of patients with more comparable covariates. The percentage of patients who required morphine titration was significantly higher in the ESPB group than in the TPVB group (74.7% vs. 38.9%, p<0.001). The observed difference between the two groups was 35.8% (95% CI [22.7%, 48.9%]). Among the patients who received morphine titration, the overall morphine doses were similar between the two groups (5.1 ml vs. 5.8 ml, p = 0.14). The results of propensity score matching analysis with the random forest model are consistent with those of the logistic regression model.
(DOCX)

S4 Table Incidence of morphine titration among centers in the MIRs03 study. ^*a*^ *Kruskal-Wallis test*. S4 Table shows the number of patients on the experimental arm who received postoperative morphine titration at the five centers in the MIRs03 study. There was no significant difference in the incidences of morphine consumption between centers in the MIRs03 study.
(DOCX)
