# Supplementary material for: Erector spinae plane block versus thoracic paravertebral block for the prevention of acute postsurgical pain in breast cancer surgery: A prospective observational study compared with a propensity score-matched historical cohort
Source: PLoS One. 2022 Dec 30;17(12):e0279648. doi: 10.1371/journal.pone.0279648 (PMC9803227; doi:10.1371/journal.pone.0279648)
Supplement: S1 Fig — Both rest and mobilization VAS peaks were encountered at 30 min of PACU stay. (DOCX) [file pone.0279648.s001.docx]

**Fig S1.** **VAS boxplot**





Fig S1 shows that both rest and mobilization VAS peaks were encountered at 30 min of PACU stay.
