# Supplementary material for: Erector spinae plane block versus thoracic paravertebral block for the prevention of acute postsurgical pain in breast cancer surgery: A prospective observational study compared with a propensity score-matched historical cohort
Source: PLoS One. 2022 Dec 30;17(12):e0279648. doi: 10.1371/journal.pone.0279648 (PMC9803227; doi:10.1371/journal.pone.0279648)
Supplement: S2 Fig — The overlapping area of propensity scores of the two groups implies that there are patients who share similar propensity scores and can thus be considered matched pairs. (DOCX) [file pone.0279648.s002.docx]

**Fig S2. Distribution of the estimated propensity scores using a logistic regression model**

**
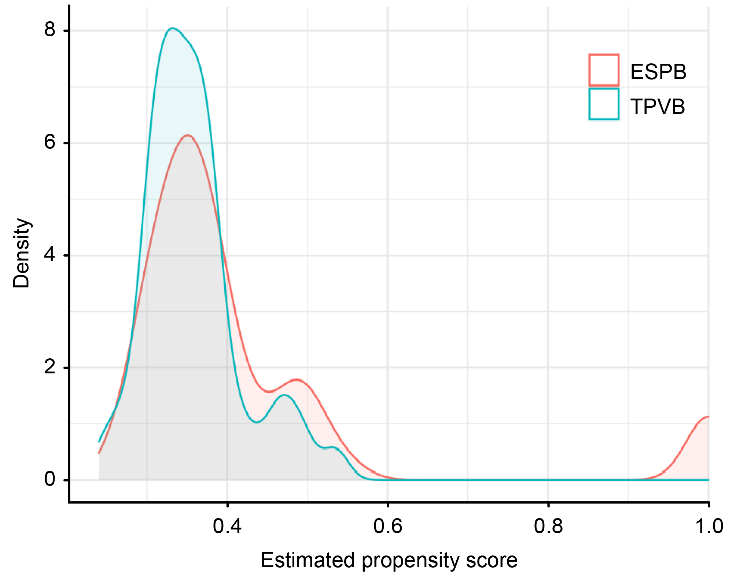
**

The overlapping area of propensity scores of the two groups implies that there are patients who share similar propensity scores and can thus be considered matched pairs.
