# Supplementary material for: Erector spinae plane block versus thoracic paravertebral block for the prevention of acute postsurgical pain in breast cancer surgery: A prospective observational study compared with a propensity score-matched historical cohort
Source: PLoS One. 2022 Dec 30;17(12):e0279648. doi: 10.1371/journal.pone.0279648 (PMC9803227; doi:10.1371/journal.pone.0279648)
Supplement: S3 Fig — presents the distributions of the estimated propensity scores using the random forest model, which are similar to those estimated by the logistic regression model in S2 Fig. (DOCX) [file pone.0279648.s003.docx]

**Fig S3. Distribution of estimated propensity scores using the random forest model**


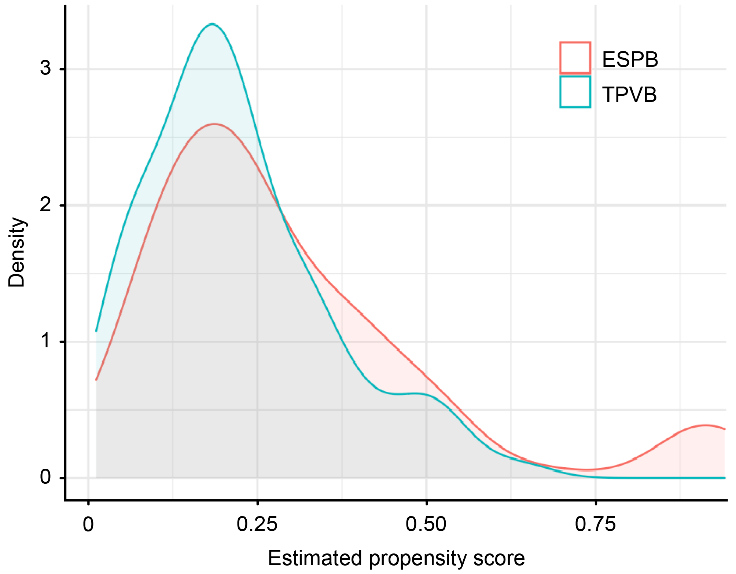


Figure S3 presents the distributions of the estimated propensity scores using the random forest model, which are similar to those estimated by the logistic regression model in Figure S2.
