# Supplementary material for: Erector spinae plane block versus thoracic paravertebral block for the prevention of acute postsurgical pain in breast cancer surgery: A prospective observational study compared with a propensity score-matched historical cohort
Source: PLoS One. 2022 Dec 30;17(12):e0279648. doi: 10.1371/journal.pone.0279648 (PMC9803227; doi:10.1371/journal.pone.0279648)
Supplement: S1 Table — There was no statistically significant difference in patient characteristics or ESPB injected volume between patients who required morphine titration and those who did not. at test when the Shapiro–Wilk test and q-q plots do not reject normality. bMann–Whitney test when the Shapiro–Wilk test or q-q plots reject normality. (DOCX) [file pone.0279648.s004.docx]

**Table S1.** **Baseline Characteristics and Injected Volume According to Morphine Titration Need Status in the ESPB Cohort.**

|  | **ESPB patients with morphine titration**  **(n = 27)** | **ESPB patients without morphine titration**  **(n = 75)** | ***P*^a,b^** |
| --- | --- | --- | --- |
| Age in years, mean (SD) | 57 (13) | 56 (13) | 0.69 |
| BMI in kg/m², median (IQR) | 24 (23-29) | 24 (22-27) | 0.21 |
| Weight in kg, median (IQR) | 67 (59-72) | 64 (55-75) | 0.38 |
| LAs volume in ml, median (IQR) | 21 (20-24) | 20 (20-26) | 0.75 |

There was no statistically significant difference in patient characteristics or ESPB injected volume between patients who required morphine titration and those who did not.

^a^t test when the Shapiro–Wilk test and q-q plots do not reject normality.

^b^Mann–Whitney test when the Shapiro–Wilk test or q-q plots reject normality.
