# Supplementary material for: Erector spinae plane block versus thoracic paravertebral block for the prevention of acute postsurgical pain in breast cancer surgery: A prospective observational study compared with a propensity score-matched historical cohort
Source: PLoS One. 2022 Dec 30;17(12):e0279648. doi: 10.1371/journal.pone.0279648 (PMC9803227; doi:10.1371/journal.pone.0279648)
Supplement: S2 Table — There was no significant difference in the need for morphine titration, overall morphine dosage or VAS at rest or mobilization according to BMI. VAS, visual analog scale. at test when the Shapiro–Wilk test and q-q plots do not reject normality. bMann–Whitney test when the Shapiro–Wilk test or q-q plots reject normality. (DOCX) [file pone.0279648.s005.docx]

**Table S2. Baseline Characteristics and Outcomes According to the Injected Volume in the ESPB Cohort.**

|  | **ESPB LAs < 25 ml**  **(n = 69)** | **ESPB LAs ≥ 25 ml**  **(n = 32)** | ***P*^a,b^** |
| --- | --- | --- | --- |
| Need for morphine titration, n | 70 | 81 | 0.32 |
| Overall morphine dosage (mg), mean | 4.7 | 6.4 | 0.05 |
| Rest VAS at 30 min, mean | 3.3 | 4.6 | < 0.01 |
| Mobilization VAS at 30 min, mean | 3.6 | 4.7 | 0.02 |
| Rest VAS at 24 h, mean | 0.8 | 1.0 | 0.62 |
| Mobilization VAS at 24 h, mean | 1.7 | 1.7 | 0.87 |
| Age in years, mean | 55.8 | 58.2 | 0.36 |
| Weight in kg, mean | 59.9 | 82.2 | < 0.0001 |

There was no significant difference in the need for morphine titration, overall morphine dosage or VAS at rest or mobilization according to BMI.

VAS, visual analog scale.

^a^t test when the Shapiro–Wilk test and q-q plots do not reject normality.

^b^Mann–Whitney test when the Shapiro–Wilk test or q-q plots reject normality.
