# Supplementary material for: Erector spinae plane block versus thoracic paravertebral block for the prevention of acute postsurgical pain in breast cancer surgery: A prospective observational study compared with a propensity score-matched historical cohort
Source: PLoS One. 2022 Dec 30;17(12):e0279648. doi: 10.1371/journal.pone.0279648 (PMC9803227; doi:10.1371/journal.pone.0279648)
Supplement: S3 Table — This table shows the comparisons of the baseline characteristics and outcomes of patients matched on propensity scores estimated by the random forest model. Ninety-five out of 102 ESPB patients were matched with a TPVB patient. Seven ESPB patients who received axillary lymph node dissection but no breast surgery were excluded from matching. Across the baseline covariates, the absolute SMDs of age and BMI were below 0.1, indicating a negligible difference. The SMD of the performed surgery type was 0.169, which slightly exceeded the preset threshold of 0.1 but was lower than the value of 0.466 before matching. The matching process created two groups of patients with more comparable covariates. The percentage of patients who required morphine titration was significantly higher in the ESPB group than in the TPVB group (74.7% vs. 38.9%, p<0.001). The observed difference between the two groups was 35.8% (95% CI [22.7%, 48.9%]). Among the patients who received morphine titration, the overall morphine doses were similar between the two groups (5.1 ml vs. 5.8 ml, p = 0.14). The results of propensity score matching analysis with the random forest model are consistent with those of the logistic regression model. (DOCX) [file pone.0279648.s006.docx]

**Table S3. Baseline Characteristics and Outcomes of Patients Matched on Propensity Scores Estimated by the Random Forest Model.**

|  | **ESPB**  **(n = 95)** | **TPVB**  **(n = 95)** | ***P*** | **SMD** |
| --- | --- | --- | --- | --- |
| Age (years), mean (SD) | 55.7 (12.9) | 56.3 (14.1) | 0.776 | 0.041 |
| BMI (kg/m²), mean (SD) | 25.1 (5.0) | 25.2 (4.5) | 0.897 | 0.019 |
| Surgery performed, n (%) |  |  | 0.510 | 0.169 |
| Mastectomy | 74 (77.9) | 78 (82.1) |  |  |
| Tumorectomy | 20 (21.1) | 17 (17.9) |  |  |
| Axillary lymph node dissection only | 1 (1.1) | 0 (0.0) |  |  |
| Need for morphine titration, n (%) | 71 (74**.**7) | 37 (38.9) | < 0.001 | - |
| Overall morphine dosage (mg), mean (SD) | 5.1 (3.0) | 5.8 (2.7) | 0.14 | - |

This table shows the comparisons of the baseline characteristics and outcomes of patients matched on propensity scores estimated by the random forest model. Ninety-five out of 102 ESPB patients were matched with a TPVB patient. Seven ESPB patients who received axillary lymph node dissection but no breast surgery were excluded from matching.

Across the baseline covariates, the absolute SMDs of age and BMI were below 0.1, indicating a negligible difference. The SMD of the performed surgery type was 0.169, which slightly exceeded the preset threshold of 0.1 but was lower than the value of 0.466 before matching. The matching process created two groups of patients with more comparable covariates.

The percentage of patients who required morphine titration was significantly higher in the ESPB group than in the TPVB group (74.7% vs. 38.9%, p<0.001). The observed difference between the two groups was 35.8% (95% CI [22.7%, 48.9%]). Among the patients who received morphine titration, the overall morphine doses were similar between the two groups (5.1 ml vs. 5.8 ml, p=0.14). The results of propensity score matching analysis with the random forest model are consistent with those of the logistic regression model.
