# Supplementary material for: Erector spinae plane block versus thoracic paravertebral block for the prevention of acute postsurgical pain in breast cancer surgery: A prospective observational study compared with a propensity score-matched historical cohort
Source: PLoS One. 2022 Dec 30;17(12):e0279648. doi: 10.1371/journal.pone.0279648 (PMC9803227; doi:10.1371/journal.pone.0279648)
Supplement: S4 Table — a Kruskal-Wallis test. S4 Table shows the number of patients on the experimental arm who received postoperative morphine titration at the five centers in the MIRs03 study. There was no significant difference in the incidences of morphine consumption between centers in the MIRs03 study. (DOCX) [file pone.0279648.s007.docx]

**Table S4. Incidence of morphine titration among centers in the MIRs03 study**

|  | **Centre 1**  **(n=120)** | **Centre 2**  **(n=7)** | **Centre 3**  **(n=25)** | **Centre 4**  **(n=3)** | **Centre 5**  **(n=10)** | ***P^a^*** |
| --- | --- | --- | --- | --- | --- | --- |
| Need for morphine titration,  n (%) | 45 (38%) | 2 (29%) | 12 (48%) | 0 (0%) | 5 (50%) | 0.5 |

*^a^ Kruskal-Wallis test*

Table S4 shows the number of patients on the experimental arm who received postoperative morphine titration at the five centers in the MIRs03 study. There was no significant difference in the incidences of morphine consumption between centers in the MIRs03 study.
